# Supplementary figures and images for: Cytomegalovirus infection in malignant pleural mesothelioma
Source: PLoS One. 2021 Aug 12;16(8):e0254136. doi: 10.1371/journal.pone.0254136 (PMC8360519; doi:10.1371/journal.pone.0254136)

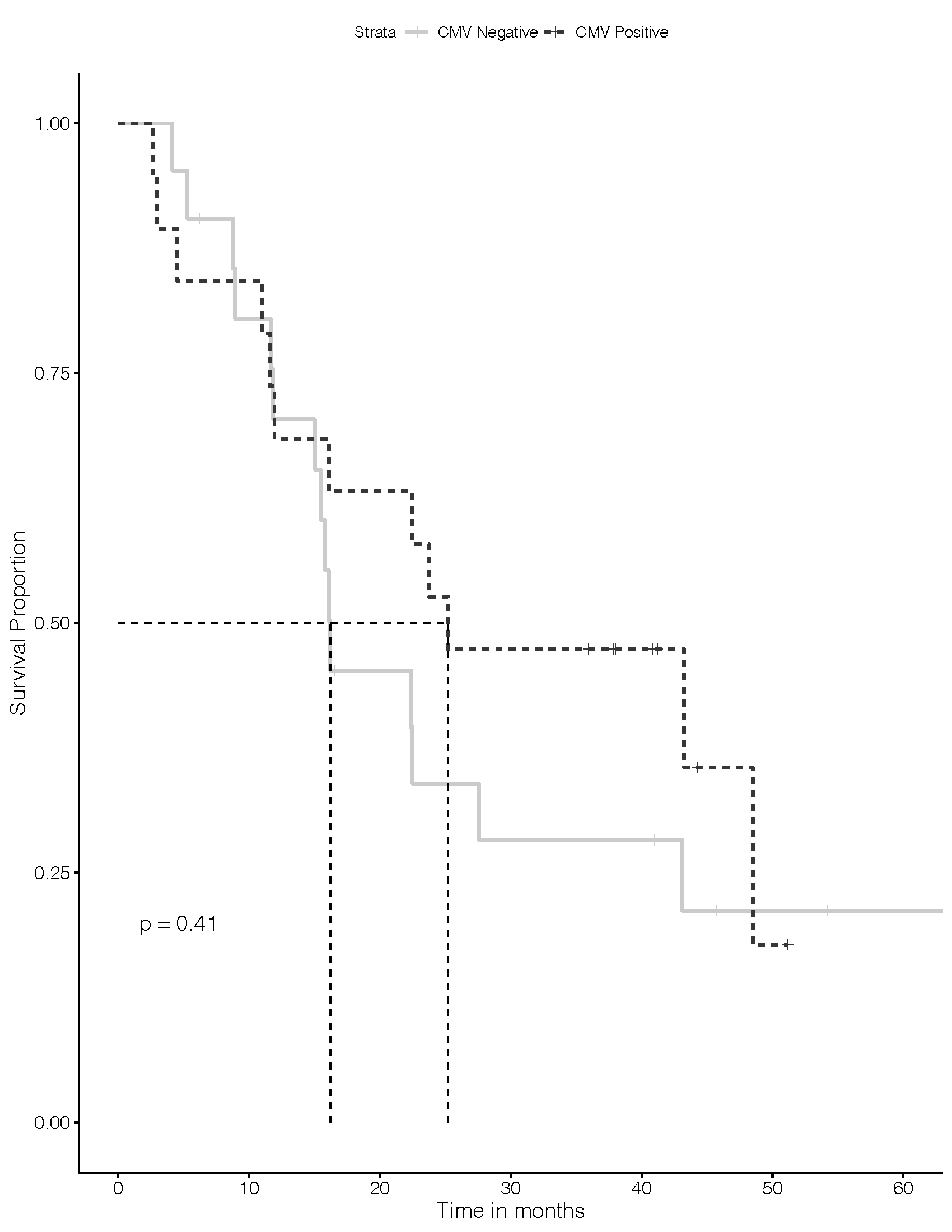

Supplement: S1 Fig — MPM patients with HCMV DNA negative tumor tissue had a shorter survival period then MPM patients with HCMV DNA positive tumor tissue. (TIF) [file pone.0254136.s001.tif]

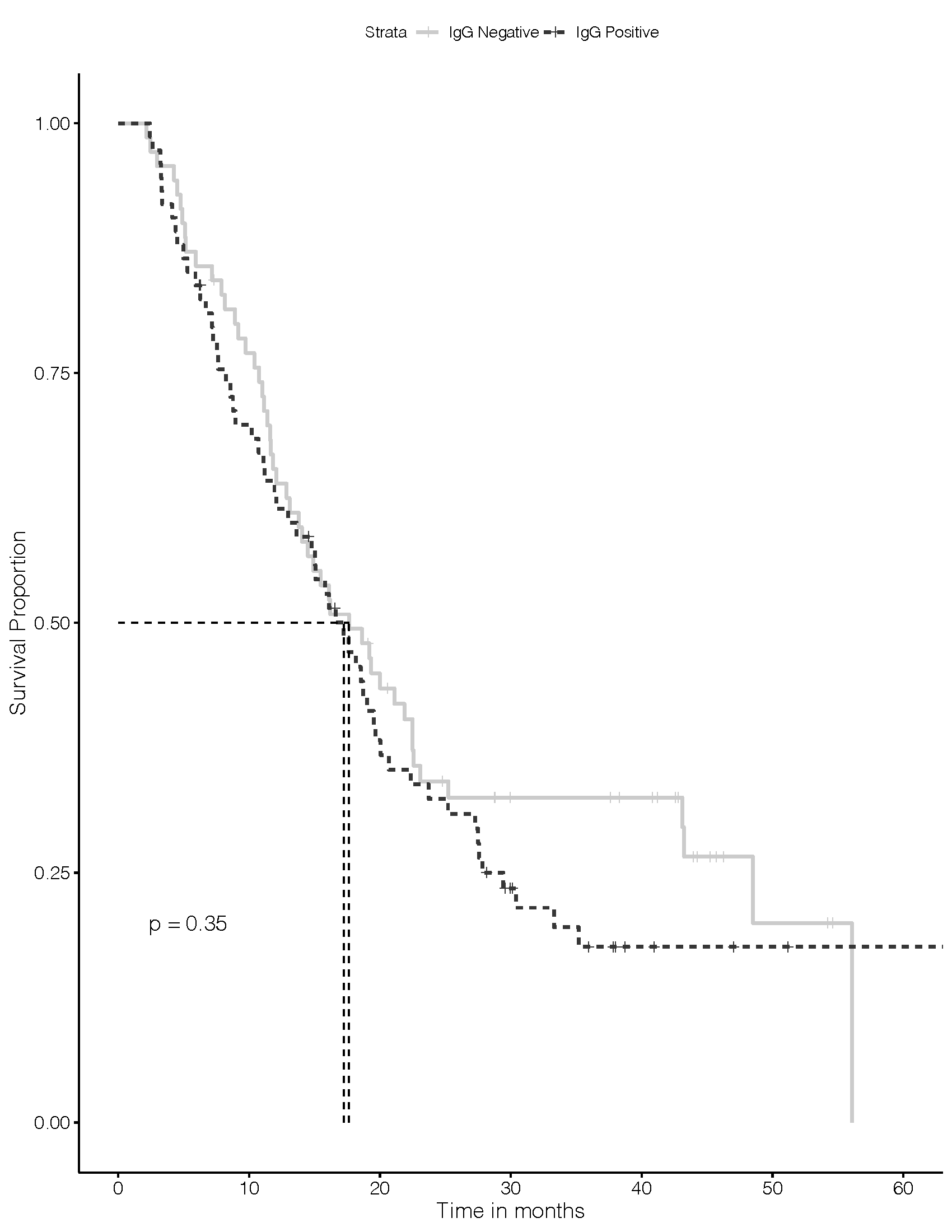

Supplement: S2 Fig — Positive HCMV IgG status, indicating the presence of HCMV antibodies, was associated with lower survival when compared to those with negative HCMV IgG status. This association was not found to be statistically significant (p = 0.35). (TIF) [file pone.0254136.s002.tif]
